# Novel perspectives on growth hormone regulation of ovarian function: mechanisms, formulations, and therapeutic applications

**DOI:** 10.3389/fendo.2025.1576333

**Published:** 2025-04-09

**Authors:** Shao Yang, Wei Luo, Yawei Sun, Shan Wang

**Affiliations:** ^1^ Graduate School, Shandong First Medical University, Jinan, Shandong, China; ^2^ Department of Reproductive Medicine, Shandong Provincial Hospital Affiliated to Shandong First Medical University, Jinan, Shandong, China; ^3^ Shandong Key Laboratory of Reproductive Research and Birth Defect Prevention, Shandong First Medical University, Jinan, Shandong, China; ^4^ Jinan Engineering Laboratory of Reproductive Diagnosis and Treatment Technology, Shandong Provincial Hospital Affiliated to Shandong First Medical University, Jinan, Shandong, China; ^5^ School of Clinical Medicine, Shandong Second Medical University, Weifang, Shandong, China

**Keywords:** assisted reproductive technology, growth hormone, premature ovarian insufficiency, follicular development, infertility

## Abstract

Delayed childbearing has led to a continuous rise in the incidence of infertility because of social development and the evolving roles of women. Assisted reproductive technology (ART) has provided new opportunities for infertility treatment, such as the application of growth hormone (GH). GH regulates ovarian function through multiple pathways, improving follicular development and hormone secretion. However, traditional GH therapy is limited by issues such as low bioavailability and insufficient delivery efficiency. In recent years, drug delivery systems based on novel biomaterials have provided breakthrough solutions for the innovative application of GH in ART. This review summarizes the mechanisms by which GH affects ovarian endocrine function and focuses on the cutting-edge advancements in GH delivery systems with examination of the innovative applications of composite biomaterials in enhancing the therapeutic efficacy of GH. By analyzing the pharmacokinetic properties of novel formulations, the safety and long-term efficacy of their clinical applications can be evaluated. GH delivery systems based on novel biomaterials considerably improve the bioavailability and targeting of GH and could lead to innovative therapeutic strategies for preventing and treating ovarian dysfunction and related diseases. By integrating multidisciplinary research findings, we provide new insights into the field of reproductive medicine that could lead to theoretical and practical importance for promoting the innovative development of ART.

## 1 Introduction

Premature ovarian insufficiency (POI) is a common reproductive endocrine disorder. It is characterized by oligomenorrhea or amenorrhea in women before the age of 40, accompanied by an elevation in gonadotropin levels (FSH > 25 IU/L). Premature ovarian failure (POF) represents the end - stage of POI, with a diagnostic criterion of FSH > 40 IU/L. Patients with POF also experience symptoms such as amenorrhea (1). According to the POSEIDON classification criteria, the assessment of ovarian reserve in female patients primarily relies on two key biomarkers: anti-Müllerian hormone levels and antral follicle count. A diagnosis of diminished ovarian reserve (DOR) is established when serum AMH concentration falls below 1.2 ng/ml or transvaginal ultrasound reveals an AFC of fewer than 5 follicles. This diagnostic criterion provides crucial quantitative parameters for clinical evaluation of female reproductive potential (2). Diminished ovarian reserve (DOR) is typically characterized by its insidious clinical presentation and lack of specific symptoms. This asymptomatic nature often results in delayed diagnosis, with most patients presenting clinical manifestations only when their ovarian function has progressed to the stage of POI.A meta-analysis published in 2019 indicated that the global prevalence rate of POI among women was 3.7% (3). POI has the following features. In POI, there is ovarian dysgenesis in which the normal developmental process of the ovaries is disrupted. Additionally, the development of ovarian follicles is impaired, and this in turn leads to a reduction in hormone secretion. Consequently, women with POI are often afflicted by infertility and menstrual disturbances (4, 5). In particular, in reproductive-aged women who have a strong desire to have genetically related children of their own, a diagnosis of POI can be devastating. Moreover, POI is closely associated with a number of other severe complications, including osteoporosis, which can cause bones to be more fragile (6) and increases the risk of fractures (7), and cardiovascular disease (8), which poses a threat to the overall health and longevity of patients. Depression is also common among patients because the physical and emotional effects of this condition can take a heavy toll on their mental health (9). At present, an effective treatment for POI remains elusive. In clinical practice, the preferred approach for treating POI is hormone replacement therapy (10). Hormone replacement therapy involves the use of estrogen or combinations of estrogen and progestin preparations. Hormone replacement therapy aims to alleviate clinical symptoms by supplementing exogenous sex hormones (11). However, this therapy has limitations because it cannot fundamentally repair the damaged ovaries. Furthermore, in patients who have fertility requirements, enhancing the ovarian response to ovulation induction medications and improving the quality of follicles are important. Therefore, there is an urgent need to investigate and develop novel strategies that can safely and efficiently restore ovarian function.

Growth hormone (GH), which is secreted by somatotropes located in the anterior lobe of the pituitary gland (12, 13), has been used for treating premature ovarian insufficiency (POI) for more than three decades. GH serves as a critical constituent within the GH-releasing hormone (GHRH)/GH/insulin-like growth factor 1 (IGF-1) axis, and it plays a major role in ovarian function (14). With regard to the specific functions of GH, it participates in multiple regulatory mechanisms within the ovaries. GH is involved in regulating steroidogenesis, which is fundamental for the biosynthesis of essential ovarian hormones such as estrogen and progesterone. Moreover, GH plays an integral part in gametogenesis, contributing to the formation and maturation of oocytes. Additionally, GH is implicated in gonadal differentiation, facilitating the proper development and specialization of the ovaries. Notably, GH can augment the sensitivity of the ovaries to gonadotropin by upregulating gonadotropin receptors, thereby optimizing the hormonal communication between the endocrine system and the ovaries.

The levels of GH and IGF-1 within follicles have been empirically demonstrated to show a positive correlation with several key aspects of reproductive processes. A considerable number of clinical studies have provided conclusive evidence showing that the administration of recombinant human growth hormone (rhGH) during the ovarian stimulation phase may reverse the age-related decline in the efficiency of assisted reproductive technology (ART) (15–17). This finding implies that GH can contribute to the improvement of oocyte and embryo quality, thereby increasing the probabilities of achieving pregnancy and live birth. This effect is particularly pronounced in patients with a poor ovarian response and those who have experienced recurrent implantation failure (18, 19). A meta-analysis in Human Reproduction Update revealed that incorporating growth hormone (GH) into controlled ovarian stimulation (COS) protocols represents an effective therapeutic approach for individuals with poor ovarian response (POR) (20). Nevertheless, despite the association between GH and POI, clinical investigations have shown certain adverse effects of GH on the risk of cancer and metabolic disorders. Additionally, issues such as sodium or water retention may occur, potentially resulting in fluid imbalance and associated clinical manifestations. Furthermore, there are concerns regarding an increased risk of breast cancer with GH treatment (22, 23). Based on the relatively extended duration of the GH treatment regimen, the potential side effects associated with it should not be disregarded (24).

Currently, the GH drugs commonly used in clinical settings generally show relatively poor stability. The half-life of rhGH ranges from only 15 to 51 minutes (25). This short half-life poses a major challenge in maintaining a stable and effective drug concentration within the body, which may consequently affect the therapeutic efficacy and safety of GH’s application. To overcome this limitation, researchers in recent years have begun to focus on combining GH with biomaterials, optimizing the release mechanism of GH through innovative delivery systems. Concurrently, researchers are improving administration methods to enhance the targeting of GH, thereby opening up new research directions for improving patients’ compliance, as well as the effectiveness and safety of treatment.

Therefore, this review comprehensively summarizes the mechanisms by which GH regulates ovarian function, encompassing its pivotal roles in modulating endocrine activity, follicular development, and ovulation processes. Specifically, we highlight the emerging therapeutic potential of GH in addressing ovarian insufficiency, particularly in the context of POF, where GH-based interventions have shown promising results in restoring ovarian function and improving reproductive outcomes. Furthermore, we provide a systematic classification and in-depth analysis of novel GH formulations based on their carrier materials, such as polymers, polypeptides, and lipids, which have revolutionized drug delivery efficiency and therapeutic efficacy. By integrating these advancements, this review aims to bridge the gap between fundamental research and clinical applications, offering innovative perspectives and actionable insights for optimizing GH use in ART. Our findings not only highlight the transformative potential of GH in reproductive medicine but also pave the way for future research and development in this rapidly evolving field.

## 2 Direct and indirect effects of GH on ovaries

### 2.1 Direct action of GH on the ovaries via GH receptor

GH directly binds to growth hormone receptor (GHR) within the ovary, directly regulating and promoting follicular growth while inhibiting follicular atresia. *GH* and *GHR* mRNA have been detected in primordial, primary, and secondary follicles of primates, where they play crucial autocrine/paracrine roles (26). GH is present in human follicular fluid, and its concentration positively correlates with oocyte quality; higher GH concentrations are associated with better oocyte quality. In human oocytes, GH binds to GHR on cumulus cells and nuclei of mature ovaries to promote nuclear maturation and the expansion of cumulus cells (27). GH is not only expressed in the ovary, but has also been detected in the placenta and uterus, suggesting that GH plays an important and unique role in female reproductive processes (28).

### 2.2 Indirect action of GH on the ovaries via IGF-1

**Figure 1:**
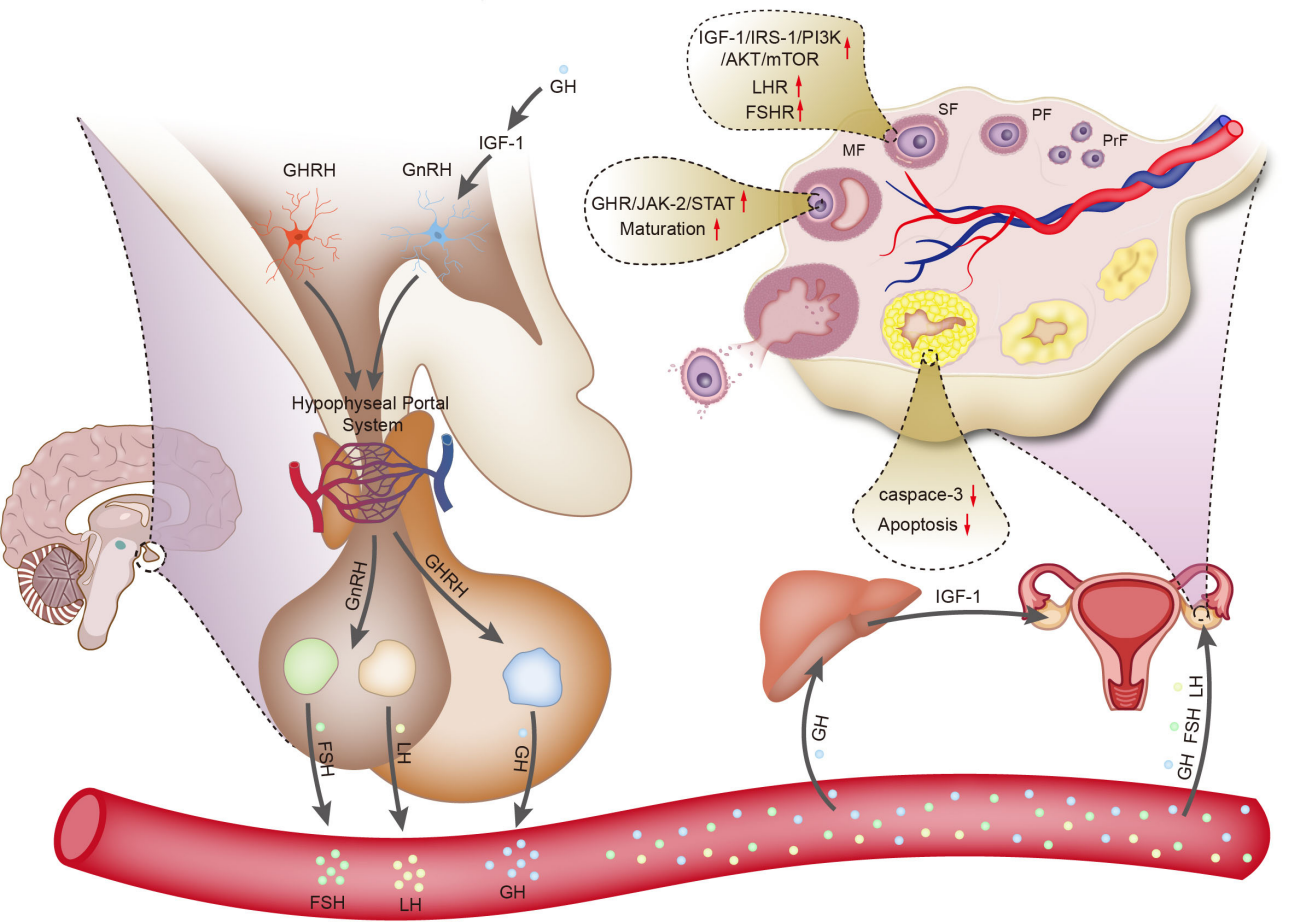
Schematic diagram illustrating the regulation of ovarian function by growth hormone.

GH acts on the liver through the circulatory system, leading to the synthesis of IGF-1. IGF-1 enters the bloodstream and acts on IGF-1 receptors in the ovary through autocrine and paracrine mechanisms, promoting the proliferation of theca cells and the replication of granulosa cells, thereby improving ovarian function (29). Studies have shown that the content of IGF-1 in granulosa cells of lambs treated with GH increases (30). Neurons in the hypothalamus responsible for producing gonadotropin-releasing hormone (GnRH) express IGF-1 receptors, indicating that gonadotropin secretion is partially regulated by the GH/IGF-1 axis. In rats, IGF-1 has dual roles. First, IGF-1 stimulates the biosynthetic activity of pituitary gonadotropin-secreting cells *in vitro*. Second, IGF-1 directly acts on GnRH neurons in rats to regulate processes related to puberty. Additionally, IGF-1 acts on kisspeptin neurons, playing a major role in regulating the activity of GnRH neurons (31) (**Figure 1**).

## 3 Mechanisms of GH in POI

### 3.1 Mechanisms of GH action on the Ovaries

In canine and bovine follicles, GH promotes the expansion of cumulus cells by stimulating cell proliferation, inhibiting apoptosis, and suppressing the synthesis of connexin43, thereby facilitating oocyte nuclear maturation (32, 33). GH of pituitary and ovarian origin can bind to GHR in the endoplasmic reticulum to form a GH: GHR complex. This complex further triggers the recruitment and phosphorylation of JAK-2 at the cytoplasmic domain of GHR. Subsequently, the GHR/JAK-2 complex induces the phosphorylation of signal transducer and activator of transcription (STAT) molecules, which translocate to the nucleus and alter gene expression and cellular activities, including cell proliferation (34). In addition to the beneficial effects of GH on nuclear maturation, it also promotes cytoplasmic maturation. This process prepares oocytes for activation and preimplantation, such as organelle redistribution, dynamic changes in the cytoskeleton, and molecular maturation (35). Brioche et al. (36) found that GH/GHR stimulated mitochondrial metabolism in older women, and this improved oocyte quality. Using single-cell RNA sequencing, Li et al. (37) found that GH promoted the development of *in vitro* matured human oocytes, with the highest oocyte maturation rate achieved at a GH concentration of 200 ng/ml. GH facilitates oocyte development by accelerating meiotic progression and regulating the cellular redox state. Further research has shown that GH upregulates the expression of genes, such as *AURKA* and *CENPE* (38). GH stimulates the liver to synthesize IGF-1, and IGF-1 is present at all stages of follicular development in ovarian cultures of sheep (39). In one study, IGF-1 was found in oocytes and granulosa cells of antral follicles and acted synergistically with follicle-stimulating hormone (FSH) to promote oocyte growth, increase the number of fully developed oocytes, and enhance the immunoreactivity of luteinizing hormone receptors (LHRs) in granulosa cells (40).

The complexity of regulatory mechanisms within the human body causes difficulty in identifying the specific roles of cytokines and signaling pathways in GH-promoted oocyte development. However, combined treatment with GH may increase granulosa cell GHR density, activate the GHR-JAK-STAT signaling pathway, and enhance the expression of transcriptional intermediates. During early follicular development, GH acts on granulosa cells to increase follicular homeostasis and development (38). When IGF-1 binds to its receptor, it phosphorylates insulin receptor substrate 1, which serves as a docking protein to facilitate the activation of phosphoinositide 3-kinase. Subsequently, the phosphoinositide 3-kinase/AKT/mammalian target of rapamycin pathway plays a crucial role in the growth and survival of granulosa cells (39). GH not only directly acts on GHR but also interacts with the endocrine system. GH combination therapy increases the density of FSH receptor and LHR in granulosa cells, enhancing their sensitivity to FSH and luteinizing hormone stimulation, thereby improving follicular development, pregnancy rates, and live birth rates (18).

The corpus luteum, which is a transient gland within the ovary that produces progesterone, undergoes dynamic changes during the menstrual cycle, and plays a pivotal role in regulating menstrual cycles and early pregnancy (41). *In vivo* studies have shown that GH transgenic mice show increased ovulation rates and litter sizes, whereas mice lacking GHR display decreased ovulation rates and litter sizes (42, 43). Protein and mRNA expression of GHR is present in the corpus luteum. Combined GH therapy also reverses the downregulation of FSH receptor, Bone Morphogenetic Protein (BMP) receptor 1B, and LHR densities before ovulation, which may improve the luteinization process following follicular maturation in patients (44). During the early stages of corpus luteum development, *in vitro* administration of GH promotes the secretion of prostaglandin F2α, supporting function of the corpus luteum (45). The supportive role of GH on the corpus luteum partly reflects its anti-apoptotic effect, and progesterone produced by the corpus luteum also serves as an anti-apoptotic factor (46, 47). GH and IGF-1 synergize with leptin to promote granulosa cell luteinization, which is initiated before ovulation. In luteal cells, mitochondrial release of Smac/DIABLO stimulates caspase-3 to induce apoptosis, while the synergistic action of GH/IGF-1 with FSH inhibits caspase-3, thereby reducing apoptosis (37).

### 3.2 Regulatory role of GH on ovarian function

GH administration, regardless of the duration (long-term and short-term), exerts positive effects on the development of preantral follicles and oocytes in prepubertal lambs, although the long-term intervention appears to be more effective than its short-term counterpart (30). Gonadotropins primarily exert their effects during the middle and late stages of follicular development. In contrast, during the early stages of follicular development, the activation of primordial follicles and their subsequent transition to primary follicles occur independently of gonadotropin regulation. This initial process is mediated through autocrine and paracrine pathways involving various growth factors (48). In the mouse model of *GHR* gene knockout, the numbers of pre-antral and antral follicles in the ovaries are lower than those in wild-type mice. This finding suggests that GH exerts a direct regulatory effect on the formation of late-stage follicles through GHR. The addition of GH to the *in vitro* culture medium of human oocytes accelerates the meiotic process, modulates the expression of genes related to cellular redox homeostasis, and enhances the developmental potential of oocytes (49). This process effectively promotes the maturation of germinal vesicle (GV)-stage oocytes after denudation. In addition to the above-mentioned effects, GH facilitates the expansion of cumulus cells by promoting their proliferation and inhibiting apoptosis. Simultaneously, under the effect of GH, cumulus cells secrete various inhibitory factors that play crucial roles in maintaining the meiotic process of oocytes and have an effect on oocyte nuclear maturation (37). GH upregulates and activates GHR on the surface of human oocyte membranes, enhancing mitochondrial activity. Mitochondria serve as key energy providers during follicular development, oocyte maturation, oocyte spindle formation, chromosome segregation, meiosis, and fertilization. Therefore, the functional status of mitochondria directly affects the formation of oocyte aneuploidy during meiosis. By increasing mitochondrial activity, GH enhances the quality and developmental potential of oocytes (50, 51). GH can improve oocyte quality by reducing oxidative stress levels in follicular fluid and granulosa cells (52).

GH directly induces steroidogenesis through a mechanism that involves increasing the mRNA expression of cytochrome P450 cholesterol side-chain cleavage enzyme. Cytochrome P450 cholesterol side-chain cleavage enzyme is a rate-limiting enzyme that catalyzes the cleavage of cholesterol side chains, thereby regulating steroid synthesis (53). GH/IGF-1 promotes the release of gonadotropins and thus regulates the function of the hypothalamic–pituitary–ovarian axis. GH/IGF-I activates the JAK/STAT and extracellular signal-regulated kinase pathways, thereby stimulating the synthesis of estradiol. Additionally, IGF-I enhances the secretion of related glycoproteins, strengthens the binding efficacy of LHR, and ultimately promotes the synthesis of progesterone (54). GH stimulates an increase in the secretion levels of estradiol and progesterone by granulosa cells (55). GH also enhances leptin-induced progesterone production in developing follicles. In rat granulosa cells, GH potentiates FSH-induced ovarian steroidogenesis by antagonizing BMP signaling (56). GH promotes the synthesis of androstenedione and testosterone in theca cells, and this effect is independent of the IGF-1 and cAMP signaling pathways (57). The above-mentioned findings indicate that sexual maturity and maintenance require appropriate GH secretion.

### 3.3 GH-mediated improvement in oocyte aging and ovarian function

The main mechanisms underlying oocyte aging are DNA damage and nuclear genome mutations. Aging further exacerbates the production of reactive oxygen species and the degree of mitochondrial DNA damage. Mice experiments have shown that GH reduces the expression level of γ-H2AX and decreases apoptosis in aged oocytes, thereby enhancing ovarian reserve capacity and oocyte quality (50). Previous studies have shown that in oocytes from patients with ovarian insufficiency, a low ovarian response, and advanced age, the number of functional mitochondria and mitochondrial DNA content are reduced and GHR expression is downregulated (58). This reduction can lead to failure in the fertilization process. The pregnancy and live birth rates are decreased in women with hypopituitarism, and GH plays an important role in enhancing fertility in these women (59). A study showed that in a group of patients aged 31–34 years, with follicles of similar size, the density of GHR on the surface of ovarian granulosa cells was decreased with declining ovarian reserve (60). In patients with a diminished ovarian reserve treated with GH, there is an increase in the density of receptors, such as GHR, FSH receptor, LHR, and BMP receptor 1B in granulosa cells, thereby enhancing their response to gonadotropins. The observed increase in LHR density in granulosa cells among patients undergoing combined GH treatment may increase sensitivity to trigger final oocyte maturation and ovulation during the human chorionic gonadotropin/LH surge. Wang et al. (52) showed that GH alleviated cisplatin-induced oxidative stress and mitochondrial damage in ovarian granulosa cells through the Sirt3-Sod2 pathway, thereby inhibiting granulosa cell apoptosis and protecting ovarian function. Cai et al. (15) studied 380 patients with a poor ovarian response. They showed that after 6 weeks of GH pretreatment, there was a considerable improvement in the oocyte use rate and embryo quality, which subsequently led to an increased live birth rate. A study by Xie et al. (61) showed that GH regulated the ovarian expression of certain genes associated with the Notch-1 signaling pathway, thereby promoting ovarian repair and regeneration in mice with premature ovarian failure. Additionally, GH induced estradiol secretion and oocyte maturation. A meta-analysis by Lin et al. (16) showed that GH had favorable clinical outcomes by improving outcomes, such as an increased number of oocytes retrieved, number of embryos obtained, embryo implantation rate, and pregnancy rate, in women with a low ovarian reserve undergoing *in vitro* fertilization.

### 3.4 Clinical application of GH in improving ovarian insufficiency

A meta-analysis published in Human Reproduction Update indicated that for patients with poor ovarian response (POR), a controlled ovarian stimulation (COS) protocol incorporating growth hormone (GH) as an adjuvant is the optimal treatment strategy. The use of GH significantly reduces the total dose of gonadotropins and improves follicular development and oocyte quality (20). However, the clinical application parameters of GH remain unstandardized, with significant variations in dosage, treatment duration, initiation timing, and discontinuation protocols. These variables may substantially influence therapeutic efficacy and safety profiles. Typically, GH is used in conjunction with COS, starting from the initiation of the COS cycle until the trigger day (hCG injection day). The timing and duration of GH administration should be individualized based on patient-specific conditions. Multiple studies have shown that a single cycle of GH use is sufficient to significantly improve follicular development and ovarian response. For example, Choe et al. demonstrated that administering three sustained-release GH injections during the mid-luteal phase, late luteal phase, and the second day of the menstrual cycle in the COS cycle increased the proportion of mature oocytes (metaphase II, MII) on the hCG trigger day compared to the control group (62). Additionally, Lee et al. further demonstrated that a single cycle of low-dose GH (10 IU, divided into three days with doses of 4 IU on the first day, 4 IU on the second day, and 2 IU on the third day) significantly improved ovarian response, the number of retrieved oocytes, clinical pregnancy rates, and ongoing pregnancy rates, particularly in patients under 40 years of age (63). Tesarik et al. found that using 8 IU of GH daily during ovarian stimulation (starting from day 7 of gonadotropin treatment until the day after hCG injection) significantly improved pregnancy outcomes in women over 40 years of age, including increased delivery rates, live birth rates, and reduced pregnancy loss (64). However, some studies have found that multi-cycle GH use can also improve IVF outcomes. For instance, Yan Gong et al. showed that starting daily administration of 4 IU of GH on day 2 of the menstrual cycle prior to ovarian stimulation and continuing until the trigger day (hCG injection day) significantly alleviated oxidative stress, improved oocyte quality, and enhanced IVF outcomes in POR patients (21). Dakhly et al. reported that initiating GH subcutaneous injections on day 21 of the preceding cycle and continuing until the hCG trigger day, in combination with a long protocol for ovarian stimulation, increased the number of mature oocytes (MII) on the hCG trigger day, as well as the total number of oocytes, fertilized oocytes, transferable embryos, and frozen embryos (65). However, long-term or multi-cycle GH use, although effective when combined with long-protocol ovarian stimulation, may significantly increase the treatment burden and potential physiological and safety risks for patients. When designing treatment protocols, it is essential to balance safety and efficacy, ensuring that the chosen regimen maximizes therapeutic benefits while minimizing patient risks. This requires clinicians to carefully evaluate the patient’s condition, treatment needs, and individual differences to determine the appropriate dosage, frequency, and duration of GH administration, aiming to optimize treatment outcomes and patient safety. Based on common clinical practices, single-cycle GH use achieves the desired effects while minimizing patient risks. In conclusion, GH is primarily used in assisted reproduction for patients with POR. Additionally, the specific molecular mechanisms by which GH increases the number of oocytes and embryos in POR patients require further investigation.

Extensive randomized controlled trial data have demonstrated that adjuvant growth hormone therapy significantly improves multiple key reproductive parameters in patients with diminished ovarian reserve, including follicular development during stimulation cycles, mature oocyte yield, fertilization success rates, and ultimately, clinical pregnancy and live birth outcomes (20, 66). The mechanism of action of GH in DOR is analogous to that observed in POI.

It is noteworthy that in patients with DOR, gas chromatography-mass spectrometry(GC-MS)metabolomics analysis identified 24 differentially expressed metabolites between the GH and control groups. As reported by He et al. (67) antioxidant metabolites, including itaconic acid and glutathione, were upregulated, while S-adenosylmethionin (SAM) levels decreased. The levels of itaconic acid positively correlated with oocyte yield, suggesting its potential role in enhancing ovarian response by reducing ROS production. Previous research has also demonstrated that derivatives of itaconic acid can attenuate ROS - induced damage. For instance, Liu et al. (68) reported that four-octyl itaconate attenuates H_2_O_2_-induced ROS production, lipid peroxidation and DNA damage. Glutathione, a powerful antioxidant, shields oocytes from oxidative damage, which may enhance their fertilization potential. According to KEGG pathway analysis, GH administration inhibits both ferroptosis and the breakdown of glutathione. Glutathione plays a critical role in various metabolic pathways, such as ferroptosis regulation, glutathione-related metabolic processes, and the synthesis of thyroid hormones. As for SAM, it participates in the metabolism of amino acids and fatty acids, as well as in DNA methylation processes, and could also play a role in the development of oocytes. As highlighted in the study by He et al. (67), GH likely modulates these metabolic pathways to optimize the oocyte microenvironment, thereby enhancing ovarian function. A 2019 retrospective study investigated patients with diminished ovarian reserve (AMH < 1.2 ng/mL), stratified into two age-based subgroups (<35 years and ≥35 years). The findings revealed that GH supplementation combined with antagonist/agonist protocols significantly enhanced live birth rates in the advanced-age group (≥35 years), with rates of 29.89% versus 17.65% (p = 0.028), demonstrating statistical significance (69). Therefore, more comprehensive studies with larger sample sizes and standardized POSEIDON criteria are required to better elucidate the adjunctive role of GH in DOR management.

Individual variability plays a crucial role in determining the therapeutic response to GH in ovarian function improvement. The study data revealed age-dependent variations in GH treatment outcomes. Regarding clinical pregnancy rates, significant improvement was observed in patients under 40 years following GH intervention, whereas this beneficial effect was absent in those over 40 years. In terms of ovarian responsiveness, GH treatment increased the number of retrieved oocytes in patients above 40 years, while its impact on younger patients remained inconclusive. Concerning gonadotropin dosage, GH administration significantly reduced the total gonadotropin requirement in advanced-age patients, whereas its effect on medication dosage in younger patients warrants further investigation (70). Research indicates that the GH/IGF-1 axis plays a significant role in the pathophysiology of polycystic ovary syndrome (PCOS), although clinical study data in PCOS patients remain controversial. Current evidence suggests that GH intervention may enhance gamete quality in PCOS patients, specifically manifested by increased oocyte fertilization rates and improved early embryonic development. While a modest improvement trend has been observed in endometrial receptivity and clinical pregnancy outcomes, these changes have not reached statistical significance (71). A clinical study demonstrated that GH supplementation significantly increased live birth rates in IVF cycles among patients with a history of poor embryonic development (72).

Research evidence demonstrates that GH administration can enhance oocyte cleavage rates across all three age cohorts (<35 years, 35-40 years, and ≥40 years) (73). Research indicates that the combination of growth hormone with controlled ovarian stimulation protocols significantly enhances assisted reproductive outcomes. This combined therapeutic approach demonstrates improved oocyte yield and maturation rates, along with enhanced fertilization success, ultimately resulting in increased availability of high-quality embryos for both fresh transfer and cryopreservation (74, 75). A retrospective cohort study involving 41 women who demonstrated suboptimal outcomes in their first IVF/PGT-A cycle, characterized by lower-than-expected MII oocyte yield, reduced blastocyst formation rate, and/or decreased euploid embryo number, revealed that growth hormone supplementation provided clinical benefits. This intervention was significantly associated with increased numbers of biopsied blastocysts and improved euploid embryo yield (76).

Therefore, when formulating individualized GH application strategies, clinicians should comprehensively evaluate patients’ baseline characteristics (including but not limited to age, ovarian reserve parameters, and body mass index) along with their previous therapeutic responses. The clinical decision-making process necessitates a careful balance between potential benefits and possible risks, accompanied by rigorous monitoring of hormonal fluctuations and adverse events throughout the treatment course.

### 3.5 Adverse effects of growth hormone administration

GH has shown certain efficacy in the treatment of diseases related to ovarian dysfunction. However, there are many side effects and safety controversies in its application, and multiple literatures have conducted research and analysis from different perspectives.

The adverse effects of growth hormone primarily encompass three major domains: tumorigenic potential, metabolic alterations, and cardiovascular risks. Several studies have shown that GH promotes the development of cancer by facilitating cell proliferation and survival, epithelial-to-mesenchymal transition, cell migration and invasion, cellular senescence, tumor growth, angiogenesis and metastasis, and drug and radiation resistance (77, 78). Research demonstrates that abnormal elevation of GH levels in adults can disrupt normal insulin signaling pathways, leading to glucose metabolism disorders. Clinical observations reveal that these metabolic abnormalities are especially prominent in acromegaly patients, who frequently exhibit marked reductions in insulin sensitivity and glucose intolerance, with some progressing to type 2 diabetes mellitus (79). Patients with acromegaly, characterized by chronic GH hypersecretion, exhibit increased cardiovascular mortality rates, frequently accompanied by hypertension, cardiac hypertrophy, and myocardial fibrosis (80). In addition, the GH–IGF1 axis is well recognized to acutely induce fluid and sodium retention and increases heart rate without a change in blood pressure (81, 82). Successful surgical resection of GH-secreting adenomas demonstrates therapeutic benefits in cardiovascular function, including improved diastolic performance, reduced left ventricular mass, and decreased heart rate and blood pressure parameters (83).

## 4 Application and advantages of novel formulations of GH

Despite the potential demonstrated by GH in the treatment of diseases related to a decline in ovarian function, the development of sustained-release formulations of GH has become important because of its short half-life, renal toxicity, and the inconvenience of frequent injections. Moreover, because of the poor targeting ability of GH, its application in the treatment of premature ovarian failure results in numerous adverse reactions. Several studies have shown that GH promotes the development of cancer by facilitating cell proliferation and survival, epithelial-to-mesenchymal transition, cell migration and invasion, cellular senescence, tumor growth, angiogenesis and metastasis, and drug and radiation resistance (77, 78). Therefore, to extend the duration of action of GH, improve drug administration methods, reduce adverse reactions, and enhance patients’ compliance, active engagement in the research and development of new paradigms for GH therapy has been performed. In this article, we have included current research advancements and summarized the new paradigms for GH treatment, aiming to provide references and insights for the development of novel GH formulations.

**Figure 2:**
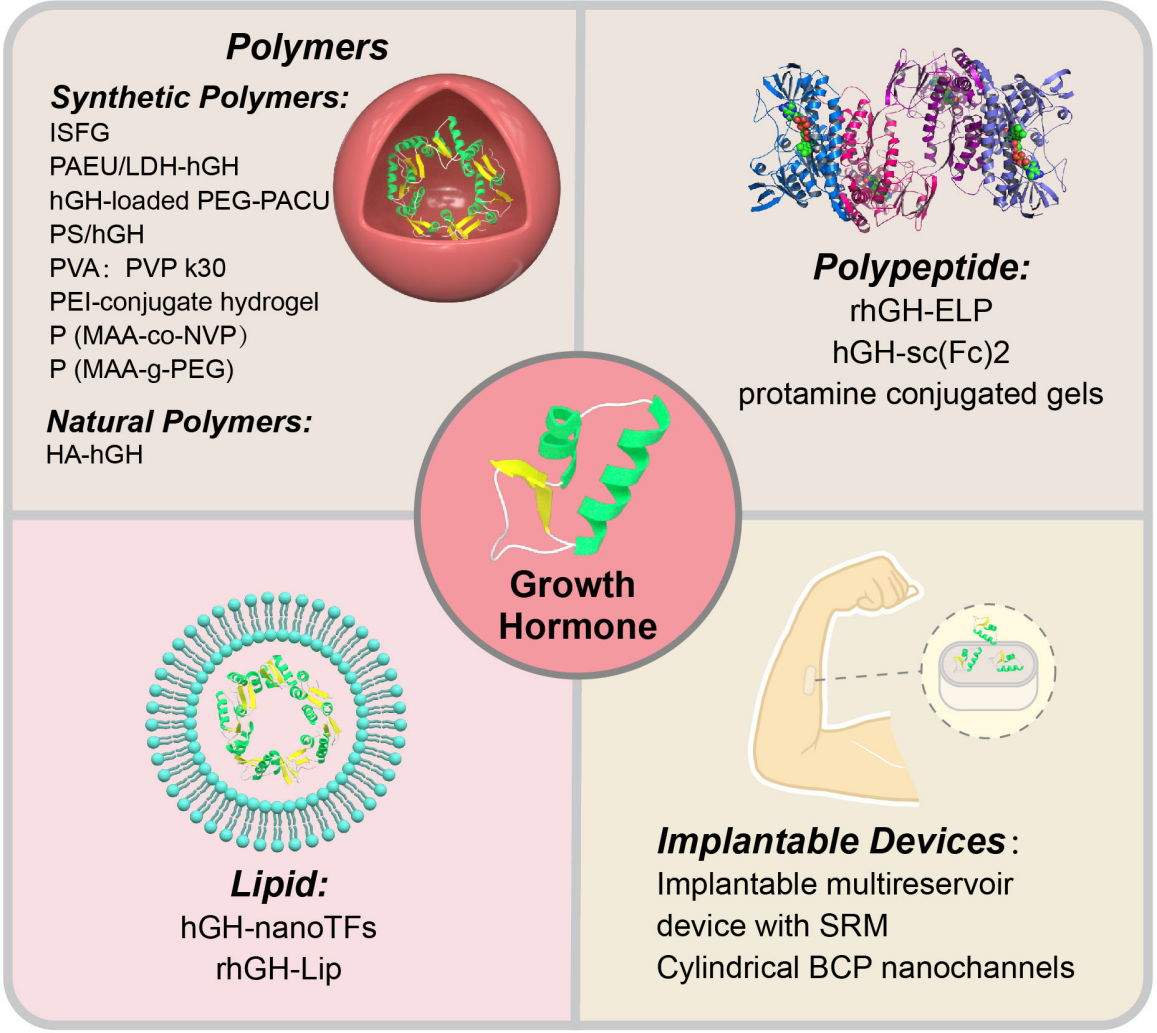
Novel growth hormone preparations.

In the research and development of novel GH formulations, researchers have primarily focused on innovations in carrier materials, with the aim of enhancing drug stability, prolonging the release time, and reducing adverse reactions by optimizing the carrier. Therefore, in this review, we elaborate on different novel GH formulations based on various carrier materials (**Figure 2**).

### 4.1 Polymer-based systems

Polymer systems, which are an important class of carrier materials, have become the focus of attention because of their excellent controllability, biocompatibility, and biodegradability. Researchers have achieved sustained and controlled release of GH by designing synthetic polymers with specific structures and functions. These synthetic polymer carriers not only effectively prolong the duration of action of GH, but also greatly enhance the patient’s convenience and comfort by reducing the frequency of injections (**Table 1**).

**Table 1:** Summary of novel growth hormone formulations loaded with a polymer carrier.

Reference	Name of new formulation	Loading growth hormone type	Release period (vitro)/(vivo)	Method of administration	Responsiveness	Cost	Production Process
Khodaverdi et al. (84)	*In-Situ* Forming Gel(ISFG)	hGH	21 days	Injectable	–	Low	Microwave-Assisted Ring-Opening Polymerization, ROP
Singh et al. (85)	PAEU/LDH–hGH	hGH	13 days/5 days	Injectable	pH/temperature	Low	Ring-Opening Copolymerization and Michael Addition Reaction
Phan et al. (86)	hGH-loaded PEG-PACU	hGH	5 days/4 days	Injectable	pH/temperature	Low	Ring-Opening Reaction and Polyaddition Polymerization Reaction
Park et al. (87)	Protamine sulfate (PS)/hGH complex	hGH	10 days/7 days	Injectable	Temperature	Low	Nucleophilic Substitution Reaction and Electrostatic Adsorption
Taheri et al. (88)	PVA:PVP k30	hGH	4+days/-	Injectable	Temperature	Low	Cold Method
Seo et al. (89)	Poly(organophosphazene)-PEI-conjugate hydrogel	–	-/4 days	Injectable	Temperature	Low	Substitution Reaction, Esterification Reaction and Amidation Reaction
Carr et al. (90)	P (MAA - co - NVP)	GH (from porcine)	45min/-	Oral	pH	Low	Free Radical Polymerization
Carr et al. (90)	P (MAA - g - PEG)	GH (from porcine)	3h+/-	Oral	pH	Low	Free Radical Polymerization
Yang et al. (91)	HA-hGH	hGH	-/10h	Patch	–	Medium	Coupling Reaction

#### 4.1.1 Synthetic polymers

Khodaverdi et al. (84) used a thermosensitive triblock copolymer of PCL-PEG-PCL to synthesize a long-acting *in situ*-forming gel for human growth hormone (hGH) injection. *In vitro* release experiments showed that the *in situ*-forming gel controlled the sustained release of hGH for up to 3 weeks. Singh et al. (85) enhanced the sustained release of hGH by dispersing LDH-hGH complexes into a cationic, pH- and temperature-sensitive, injectable poly PAEU copolymer hydrogel through dual ionic interactions. The synthesized nanobiohybrid hydrogel (PAEU/LDH–hGH) showed an extended-release period of 13 days *in vitro* and 5 days *in vivo*. Phan et al. (86) designed a novel injectable formulation of a PEG-PACU copolymer loaded with hGH. This formulation showed good biocompatibility and sustained release of hGH for 5 days *in vitro* and 4 days *in vivo*. The formulation formed a gel under physiological conditions (pH 7.4, 37°C). After injection into the back of rats, this formulation formed a gel with a porous structure that allowed for sustained release of hGH. Notably, no major tissue inflammation was observed around the injection site. Park et al. (87) developed a dual ionic interaction system consisting of a cationic complex (polyelectrolyte complex) containing hGH and an anionic thermosensitive hydrogel. This composite system showed sustained release of hGH for 10 days *in vitro*. In hypophysectomized rats, a single injection of polyelectrolyte complex-loaded anionic hydrogel resulted in a higher growth rate than daily injections of hGH solution over 7 days, which indicated the effective sustained release of active hGH. Taheri et al. (88) developed a thermosensitive and biodegradable PVA-PVP k30-poloxamer 407 hydrogel. *In vitro* experiments showed that this hydrogel system was in a liquid state below the normal body temperature and transformed into a semi-solid transparent gel at the normal body temperature. By adjusting the ratio of PVA and PVP k30, the concentration of poloxamer 407 could be reduced, thereby lowering the risk of adverse reactions. *In vitro* studies have shown that this hydrogel enables sustained release of GH, mitigating the initial burst release of GH. Seoet al. (89) developed an injectable, ionic, thermosensitive poly(organophosphazene)-polyethyleneimine conjugate hydrogel. *In vitro* experiments showed that this hydrogel inhibited the initial burst release of GH and extended its release period. Compared with non-ionic hydrogel or GH solution controls, this hydrogel increased the area under the curve of the pharmacokinetic profile. *In vivo* experiments showed sustained release of GH from the hydrogel. In a hypophysectomized rat model, based on indicators of body weight gain and tibial growth plate width, the bioefficacy of GH released from this hydrogel was similar to that of daily administration over 4 consecutive days. Carr et al. (90) developed a complexing hydrogel based on poly(methacrylic acid-co-N-vinylpyrrolidone) (P(MAA-co-NVP)). *In vitro* experiments showed that the P(MAA-co-NVP) hydrogel system was capable of inhibiting the initial burst release of GH and extending the duration of drug release. At a pH of 7.4, P(MAA-co-NVP) microparticles achieved 90% GH release within 45 minutes compared with another system, P(MAA-g-PEG) microparticles, which required 180 minutes to reach the same proportion of release. Additionally, under gastric acid conditions (pH 1.2), P(MAA-co-NVP) microparticles showed minimal drug release, which demonstrated their stability in the stomach, which helped to avoid drug loss before reaching the small intestine. Compared with traditional subcutaneous administration, the major advantage of this hydrogel system is its oral delivery, enhancing the patient’s convenience and reducing the pain and discomfort associated with injections.

#### 4.1.2 Natural polymers

Yang et al. (91) synthesized a hyaluronic acid (HA)-hGH conjugate through a specific conjugation reaction between aldehyde-modified HA and the N-terminal amino group of hGH. Keratinocytes and fibroblasts in the skin possess HA receptors, and fibroblasts also have hGH receptors. Using the receptor–ligand binding properties, this complex can facilitate the transdermal delivery of protein-based drugs. *In vitro* experiments showed that the HA-hGH conjugate maintained the immunogenicity of hGH compared with hGH alone (67). The biological conjugation of hGH with HA effectively reduced renal clearance and enzymatic degradation *in vivo*, thereby enhancing the bioavailability of hGH.

### 4.2 Elastin-like polypeptide

The protein–peptide system, which is an innovative drug delivery platform, shows efficacy in extending the duration of action of GH through an optimized carrier design. Yang et al. (92) developed an ultra-long-acting GH-polypeptide fusion protein for the treatment of GH deficiency. They found that elastin-like polypeptide extended the plasma half-life of rhGH from 0.7 hours to 594.6 hours. They also showed that a single subcutaneous injection of rhGH-elastin-like polypeptide continuously promoted linear growth and the development of major tissues and organs in rats for 3 weeks. Hematoxylin and eosin staining of major organs in rats showed that rhGH-elastin-like polypeptide had good biocompatibility. All of these findings were achieved by sustained release to prolong the duration of action of GH.

Zhou et al. (93) designed a novel Fc-based drug carrier called the single chain Fc-dimer (sc(Fc)2). The sc(Fc)2 consists of two Fc domains recombinantly linked through a flexible linker, thereby omitting the hinge region to further stabilize it against proteolysis and reduce FcγR-related effector functions. Using sc(Fc)2 as a protein drug carrier, *in vitro* release experiments showed that this novel carrier protein had a longer *in vivo* half-life and higher hGH-mediated bioactivity than traditional Fc-based drug carriers. The bioactivity of hGH was assayed using Nb2 cell proliferation experiments, which showed similar bioactivity between free-form hGH and hGH-sc(Fc)2. *In vivo* release experiments in male CF1 mice showed that the half-life of hGH-sc(Fc)2 was extended compared with hGH-Fc, by approximately double, and greatly exceeded the previously reported half-life of hGH in male CF1 mice (< 15 minutes). Additionally, monitoring changes in IGF-1 levels further validated the *in vivo* bioactivity and prolonged pharmacodynamics of hGH-sc(Fc)2. Compared with previous studies, the main advantage of this research is the design of a more stable and efficient single-chain Fc dimer (sc(Fc)2) as a drug carrier. This drug carrier not only overcomes issues, such as physical instability and reduced bioactivity present in traditional Fc fusion protein drugs but also extends the half-life and enhances the bioactivity of hGH. Therefore, sc(Fc)2 potentially provides a novel strategy and method for improving the pharmacokinetics and bioactivity of protein drugs. Park et al. (94) developed a cationic and thermosensitive protamine-conjugated poly(organophosphazene) hydrogel for sustained human growth hormone (hGH) delivery. This system leverages electrostatic interactions between negatively charged hGH and cationic protamine, enabling prolonged release. The hydrogel undergoes sol-gel transition at 37°C, facilitating easy injection and *in vivo* gelation. *In vitro* studies showed suppressed burst release and extended hGH release up to 7 days, controlled by hydrogel degradation. *In vivo* studies in rats and monkeys demonstrated extended hGH half-life (21.9 hours in rats, 23.6 hours in monkeys) and sustained IGF-1 elevation for 13 days, comparable to daily injections. Biocompatibility was confirmed via gel retardation assays and SDS-PAGE, showing native hGH release without aggregation. This system outperforms traditional PLGA-based methods by combining thermosensitivity and cationic properties, enhancing stability, reducing injection frequency, and improving patient compliance (**Table 2**).

**Table 2:** Summary of novel growth hormone formulations loaded with polypeptide carriers.

Reference	Name of new formulation	Loading growth hormone type	Release period (vitro)/(vivo)	Method of administration	Responsiveness	Cost	Production Process
Yang et al. (92)	rhGH-ELP	rhGH	/594.6 h	Injectable	–	High	Genetic Engineering
Zhou et al. (93)	hGH-sc(Fc)2	hGH	4h/4h	Injectable	–	High	Genetic Engineering
Park et al. (94)	Protamine conjugated gels	hGH	7days/21.9 h in rats, 23.6 in monkeys	Injectable	–	High	Substitution Reaction, Deprotection Reaction and Amidation Reaction

### 4.3 Lipids

Lipids, as carrier materials, have considerable advantages in drug loading. The high biocompatibility and degradability of lipids reduce drug irritation and toxicity, thereby enhancing safety and effectiveness. Lipid carriers have a high encapsulation efficiency and drug loading capacity, enabling efficient loading and stable delivery, which improves drug use. In the delivery of protein drugs such as GH, lipid carriers enhance stability and bioavailability, thus improving patients’ convenience. Therefore, lipids play a crucial role in drug delivery. Kateh Shamshiri et al. (95) designed lecithin-soya phosphatidylcholine nanotransfersomes, which is a complex that serves as a potential carrier for the transdermal administration of hGH. These are highly flexible vesicles known as transfersomes. *In vitro* experiments by Kateh Shamshiri et al. (95) showed good stability of this complex, with well-preserved hGH. When hGH was applied to excised rat skin, it began to gradually penetrate the skin after half an hour and could be sustainedly released for longer than 10 hours. The use of these nanotransfersomes loaded with hGH for transdermal delivery reduces patients’ discomfort, enhances patients’ compliance, and prolongs the duration of drug action because of sustained release.

Taghizadeh et al. (96) designed a novel nanoliposomal system capable of stably encapsulating GH within liposomes through nanotechnology and investigated the preventive effects of topically applied rhGH-Lip on ultraviolet B-induced damage. This formulation remained stable for more than 1 year when stored at 4°C. The biocompatibility and safety of this novel formulation were verified using Ba/F3-hGHR, HaCaT, and HFFF-2 cell lines. *In vitro* release experiments showed that this nanoliposomal formulation enhanced the transdermal absorption of hGH and achieved a substantial increase (> 10 fold) in transdermal delivery efficiency compared with its free form. Further validation through nude mouse experiments confirmed this formulation’s effectiveness in preventing ultraviolet B-induced skin damage and showed its potential for anti-photoaging and anti-wrinkle effects. The main advantage of this study is the successful development of a novel nanoliposomal system for encapsulating and topically delivering hGH. This system not only improved the skin permeability of GH but also confirmed its effectiveness in preventing ultraviolet B-induced skin damage through animal experiments. Additionally, this formulation may provide a new strategy and method for the dermal delivery of other protein drugs (**Table 3**).

**Table 3:** Summary of novel growth hormone formulations loaded with lipid carrier.

Reference	Name of new formulation	Loading growth hormone type	Release period (vitro)/(vivo)	Method of administration	Responsiveness	Cost	Production Process
Maryam Kateh Shamshiri et al. (95)	hGH‐nanoTFs	hGH	10h+/10h+	Patch	–	High	Modified Thin-Film Hydration Method
Bita Taghizadeh et al. (96)	rhGH-Lip	rhGH	–	Dermal topical administration	–	High	Fusion Method

### 4.4 Implantable devices for controlled growth hormone delivery

Implantable devices exhibit significant advantages in the medical field, particularly in the area of controlled drug release. These devices are capable of precisely regulating the release rate and dosage of medications, maintaining a stable concentration of drugs in the body. This precise controlled-release technology enhances therapeutic efficacy, minimizes drug wastage, and reduces potential side effects associated with fluctuations in drug concentration. Implantable devices enable long-term or periodic sustained drug administration, eliminating the need for frequent injections or oral medications. For patients requiring long-term subcutaneous or intramuscular injection of growth hormone therapy, this significantly improves the convenience of drug use and enhances patient compliance.

Lee et al. (97) designed an implantable multi-reservoir device with a stimulus-responsive membrane for on-demand and pulsatile delivery of GH. This device releases hGH through non-invasive near-infrared (NIR) irradiation from the external skin, with no drug release occurring during non-irradiation periods. These authors showed that this device sustainably released hGH for more than 30 days *in vitro*. Using hypophysectomized rats, the pharmacokinetic and pharmacodynamic characteristics of the device were evaluated after daily NIR irradiation for 14 days. Rats in the experimental group were implanted with the device and underwent daily NIR irradiation to release GH, while rats in the control group received intermittent subcutaneous injections of GH in a conventional manner. Serum IGF-I concentrations were used as a pharmacodynamic marker for hGH administration. The authors showed that serum IGF-1 concentrations in both groups of hypophysectomized rats were similar, which indicated that the device achieved controlled release of GH. Hematoxylin and eosin staining of the local subcutaneous tissue in rats showed that continuous NIR irradiation did not cause damage to the subcutaneous tissue, and similar to other implants, mild inflammatory reactions occurred around the implant site.

Seung Yun et al. (98) developed a single-channel diffusion device for protein drugs mediated by cylindrical nanochannels. *In vitro* experiments showed that this device achieved sustained and controlled release of hGH by precisely controlling the pore size of the nanochannels. When the diameter of the nanochannels was less than twice the hydrodynamic diameter of the protein drug, the protein drug molecules could only pass through the channels in a single-molecule manner, thereby enabling precise regulation of the release rate. Additionally, the device showed good stability and durability in *in vitro* release tests, with the nanoporous structure and gold deposition layer maintained over a test period of up to 2 months. *In vivo* experiments showed that after implantation of the device in rats, sustained release of GH was achieved with a stable release profile, and the released GH maintained good biological activity. The device provided a longer duration of drug action than that with direct injection of GH, reducing the need for frequent injections (**Table 4**).

**Table 4:** Summary of novel growth hormone formulations loaded with implantable devices.

Reference	Name of new formulation	Loading growth hormone type	Release period (vitro)/(vivo)	Method of administration	Responsiveness	Cost	Production Process
Seung Ho Lee et al. (97)	Implantable multireservoir device with SRM	hGH	/14+ days	Implantable device (subcutaneous)	Near-infrared (NIR) light	High	Polymerization Reaction, Oxidation Reaction and Reduction Reaction and Hydrothermal Reaction
Seung Yun et al. (98)	Cylindrical BCP nanochannels	hGH	2 months+/3 weeks	Implantable device (subcutaneous)	–	High	Anionic Polymerization Reaction

## 5 Conclusions and future perspectives

In recent years, the pivotal role of GH in ovarian health and function has increasingly received considerable attention from the scientific community. GH not only markedly promotes follicular development and enhances overall oocyte quality but also plays an indispensable role in optimizing the ovarian microenvironment and precisely regulating a balance of the endocrine system. These groundbreaking discoveries have provided a novel perspective for investigating effective treatment strategies for the complex conditions of DOR or POI. A key breakthrough of this study lies in being the first to link modern, novel GH formulations with POI, thereby bolstering confidence in the application of GH in assisted reproductive technologies. Using cutting-edge pharmaceutical formulation research, scientists are actively engaged in investigating innovative carriers and delivery methods, aiming to substantially enhance the bioavailability of GH and prolong its sustained release efficacy. The innovative application of nanotechnology, particularly the ingenious design of nanoparticles and nanochannels, has led to a revolutionary breakthrough in precise GH delivery, enabling more accurate and efficient targeted transport. Furthermore, the clever integration of biomaterials with intelligent drug delivery systems allows for automatic adjustment of GH release rates in response to subtle changes in the internal environment, thereby further enhancing therapeutic outcomes. However, the practical application of GH in the field of DOR or POI still requires more in-depth investigation and research, such as comprehensive determination of its mechanism of action, continuous optimization of delivery strategies, and extensive performance of large-scale clinical trials. The combined application of GH with other advanced therapies may offer unprecedented innovative solutions for the treatment of DOR and POI. Despite these advancements, it is important to acknowledge the limitations of this study. Given the differences in terminology and research.
